# The effect of quality of service experience on consumers' loyalty to music streaming services: Time pressure as a moderator

**DOI:** 10.3389/fpsyg.2022.1014199

**Published:** 2022-11-11

**Authors:** Yizhou Zhang, Mengze Zhang

**Affiliations:** ^1^Department of Global Business, Anyang University, Anyang, Gyeonggi-do, South Korea; ^2^Department of Economics, Sejong University, Seoul, South Korea

**Keywords:** quality of service experience, music streaming services, time pressure, moderator mediation model, loyalty

## Abstract

This study investigates how the quality of service experience (QSE) impacts users' satisfaction and loyalty to music streaming services. To this end, the sense of insufficient time to do things, a moderated mediation model, is adopted to examine the mediating role of satisfaction and the moderating role of time pressure from working. By using structural equation modeling, the results reveal that QSE is positively related to users' satisfaction and loyalty to music streaming services. The results also show that the QSE positively influences users' loyalty through satisfaction. Furthermore, time pressure, acting as a moderator, positively affects the relationship between QSE and satisfaction and the relationship between satisfaction and loyalty.

## Introduction

Since our consumption determines our standard of living, expenditure on music is integral to cultivating our lives (Kim and Kang, 2022). Notwithstanding the emergence of new technologies and the initiation of the digital age (Arditi, 2013), consumer demand for music remains unchanged (Chang et al., 2021). There has been a consistent decline in industry-wide revenue from physical music sales, from $ 23.3 billion in 2001 to only $ 5.0 billion in 2021 (International Federation of the Phonographic Industry, 2022), while music streaming has begun to capture the market, accounting for only $ 0.1 in 2005 and growing to $ 16.9 billion by 2021, constituting 65.0% of recorded music revenue. Consumers' paid subscription to music streaming services boosted the industry's overall growth (International Federation of the Phonographic Industry, 2022). In other words, music streaming platforms are considered facilitators of the spread of music, using music streaming services that allow consumers to choose and enjoy their personal music preferences. Enjoying music can be considered an experience that may influence consumers' feelings and thinking about the music streaming service. Therefore, it is imperative to know whether these experiences and feelings will influence consumers' opinions of the platforms and their decisions to continue using and subscribing to them.

Because of its large population, China's music market ranked sixth in the global market after the US, Japan, the UK, Germany, and France (International Federation of the Phonographic Industry, 2022). Specifically, there are 680.98 million music-streaming users in China, accounting for about 67.4% of all Internet users (China Internet Network Information Center, 2022). Therefore, streaming can be regarded as the preferable way to listen to music since the use of smartphones with internet access is ubiquitous (Kim et al., 2017). However, it remains unclear how music streaming platforms that only provide services in Chinese, like QQ, could get more profits and maintain long-term gains. Hence, music streaming platforms must be aware of consumers' motivations for continuing to use and subscribe to these streaming services.

The revenue generated from the enormous consumer base is noteworthy. According to Tencent Music Entertainment Group's Full Year 2021 Unaudited Financial Results, at the end of 2021, revenues for online music services rose 22.7% to RMB 11.47 billion ($ 1.80 billion) in 2021, up from RMB 9.35 billion during the same period of 2020. This increase was attributed to a growth in music subscription revenue of RMB 7.33 billion ($ 1.15 billion) although revenue generated by social entertainment services and others declined modestly by 0.1% to RMB 19.78 billion ($ 3.1 billion) in 2021 from RMB 19.80 billion in 2020 due to greater competition stemming from other pan-entertainment platforms and the impact of the macro environment change this was decreased by revenue growth from live audio. Based on this data, music streaming platforms can be regarded as the premier application for enjoying music and participating in entertainment activities, mainly owing to the emergence of Web 2.0 and social networking services applied to music streaming platforms.

One issue encountered by music streaming platforms is that they have provided value-added services besides playing music, which faces rising competition (Hagen and Lüders, 2017). More specifically, in these virtual communities, consumers can comment and express their thoughts and feelings about songs they are listening to, interact based on others' comments, and also participate in “groups” with similar music (Nguyen et al., 2014; Weinberger and Bouhnik, 2021). In addition, at the end of the year, music streaming platforms can provide consumers with a report about their respective music playing histories with user authorization, including the total number of songs listened to and the total length of time over the year, and the most frequently listened music and singers, which becomes a music database (Kreitz and Niemelä, 2010; Weinberger and Bouhnik, 2021). In marketing competition, it is essential for this type of research to determine whether virtual social services and interaction with consumers influence satisfaction and loyalty to music streaming platforms. However, this latest consumer experience is newly available, meaning that the amount of information around this theme is not yet rich and well-systematized. Few studies on the satisfaction and loyalty to music streaming services have considered virtual communities and interactions.

In modern society, human beings face acute time pressure. As a result, time pressure is known to influence working behavior (such as working efficiency, teamwork performance, and management decisions) in the accounting and audition fields (Svanström, 2016; Rostami et al., 2019; Bjorvatn and Wald, 2020; Ewing and Spilker, 2021). Furthermore, in the retail industry, time sensitivity turned out to be a significant factor affecting their choice (Hussein and Kais, 2020). Specifically, at duty-free stores, the time pressure of the shopping process reinforces negative emotions, leading to an increase in emotional impulse purchases (Sohn and Lee, 2016), and time pressure influences the relationship between purchase motivations and choice of commercial activities (Lin and Chen, 2013). However, in the field of music streaming services, there is less existing research studying whether people's time pressure from working affects the relationship between their mental experience and loyalty.

For the consumption of music streaming, some existing studies analyzed the factors influencing subscriptions based on the theories of psychology (such as consumers) and others analyzed it based on subscription price and platform design (Danckwerts and Kenning, 2019; Kinnally and Bolduc, 2020; Barata and Coelho, 2021; Chang et al., 2021). It was found that attitudes, social interactions, and injunctive norms are indicative predictors of people's willingness to subscribe to music streaming services (Bolduc and Kinnally, 2018; Kinnally and Bolduc, 2020; Chang et al., 2021). In addition, price value, habit, and performance expectancy play a dominant role in shaping the willingness to pay for music streaming services (Barata and Coelho, 2021; Lüders, 2021). Furthermore, even music-based psychological ownership is closely associated with consumers' willingness to choose paid services (Danckwerts and Kenning, 2019).

Although previous efforts seek to better understand whether the social function of a streaming platform influences consumers' purchase behaviors, two aspects have been ignored: the artistic element of music and the social function of music streaming platforms that satisfies users' demands for mental relaxation and social interaction. The quality of service experience (QSE) includes four factors namely, hedonics, peace of mind, involvement, and recognition (Otto and Ritchie, 1996; Cervera-Taulet et al., 2019; Schlesinger et al., 2020), which could measure consumers' spiritual enjoyment with using music streaming services.

Thus, this study aims to clarify the spiritual experience measured by the influence of QSE on users' satisfaction and loyalty. It aims to explore the effect of QSE on users' satisfaction with and loyalty to music streaming platforms that do not provide music appreciation but various social activities, particularly to understand users' subjective assessment and willingness to continue subscribing when facing time pressure from work. This could foster consumer value and ensure sufficient profitability for the industry.

The study seeks to achieve the objective by introducing a second-order model of QSE created by Otto and Ritchie (1996) and using the construct of time pressure as a moderator. Furthermore, the study only considers consumers who subscribe to music streaming services, allowing us to examine the direct impact of QSE and loyalty and the impact of time pressure on the relationship between satisfaction and loyalty.

This paper is structured as follows. First, the conceptual framework is presented, and hypotheses for the model are developed. Then, a quantitative analysis is conducted using primary data on users' perceptions of music streaming services. Next, the empirical work and a discussion of the results, as well as implications for both theory and practice, are provided. Finally, the current study's limitations and possible further research interests are discussed.

## Literature review and hypotheses

### QSE

Experience embodies one's perception and interpretation of one or more events (Brunnström et al., 2013). For instance, the experience might be a result of human encounters with systems, services, or artifacts (Brunnström et al., 2013). Experience quality (or quality of experience) is the extent to which a user is pleasured or annoyed by an application or service, also known as QSE (Brunnström et al., 2013). Quality of service experience is based on the concept of “service experience” created by Otto and Ritchie (1996), also called experience quality (Cervera-Taulet et al., 2019; Schlesinger et al., 2020). As the affective component of the experience, experience quality consists of subjective, emotional, and personal responses to various aspects of service development, resulting in overall satisfaction. The QSE construct initially included six dimensions: hedonic, interactive, novelty, comfort, safety, and stimulation (Otto and Ritchie, 1995). These were further explicated into four dimensions: hedonics, peace of mind, involvement, and recognition (Otto and Ritchie, 1996). Hedonics is defined as a set of emotional reactions associated with pleasure, enjoyment, the desire to experience various things, and sharing with others (Cervera-Taulet et al., 2019; González-Rodríguez et al., 2019; Schlesinger et al., 2020). Peace of mind refers to the need for relaxation, comfort, security, and privacy of body and mind (González-Rodríguez et al., 2019; Suhartanto et al., 2019; Schlesinger et al., 2020). Involvement comprises the area of participation, education, decision-making, and control during the tour (Cervera-Taulet et al., 2019). Finally, recognition refers to the emotional response to a tour that results from feeling important or considered in the experience (Schlesinger et al., 2020).

Several recent studies also adopted Otto and Ritchie (1996)'s approach to assessing QSE in the tourism industry (Cervera-Taulet et al., 2019; Ghorbanzadeh et al., 2020; Schlesinger et al., 2020). Specifically, there is much research that discusses the influence of QSE on consumers' satisfaction and loyalty in emerging Mediterranean destinations (Cervera-Taulet et al., 2019; Schlesinger et al., 2020), creative tourism (Suhartanto et al., 2019), dark tourism (Ghorbanzadeh et al., 2020), and heritage tourism (González-Rodríguez et al., 2019). Through these studies such as in heritage tourism, it was found that the quality of experience positively and directly impacts satisfaction (González-Rodríguez et al., 2019). Also, in emerging Mediterranean destinations, QSE directly and positively affects loyalty (Schlesinger et al., 2020) and revisit intention (Cervera-Taulet et al., 2019). Especially in creative tourism and dark tourism, experience quality is a crucial determinant of tourist loyalty (Suhartanto et al., 2019; Ghorbanzadeh et al., 2020). Similarly, in the business field, the service experience is considered to have a greater impact on satisfaction, and service experience-driven satisfaction showed a positive effect on loyalty (Roy et al., 2019). Hence in the field of streaming services, there is much research studying the relationship between QSE and satisfaction and both QSE and loyalty (Baraković et al., 2020).

It is revealed that service consumption experience does not directly influence consumer satisfaction in the electronically mediated environment (Dai and Salam, 2019). On the other hand, QSE impacts users' intentions to keep and order streaming services in the future, however, the association between satisfaction and intentions to keep and order streaming services weakens with habit strength (Gupta and Singharia, 2021). Therefore, some researchers have studied the influence of consumers' satisfaction on loyalty (or willingness to continue to subscribe) in the streaming services domain (Kondo et al., 2010; Khatib et al., 2019; Hsu et al., 2021). The user behavior-satisfaction-loyalty model is established to explain the trajectory of influence on loyalty and repurchase intention (from satisfaction to intention to continued future use) (Kondo et al., 2010) and can be predicted by consumer satisfaction (Khatib et al., 2019; Hsu et al., 2021).

### Satisfaction

A well-studied concept in the field of marketing, satisfaction refers to a summative mental state when emotions surrounding uncertain expectations are combined with a consumer's previous feelings about their consumption experience (Oliver, 1980). On the other hand, Oliver proposed that satisfaction resulted from the customer's practical experience with the product and service compared to their expectations of it. Both cognitive judgments and affective experiences are also agreed to be critical in generating satisfaction (Oliver, 2014). Also, satisfaction can be described as the result of ongoing processes by which consumers evaluate products or services by comparing their expectations, prior experience, and current usage (Anderson, 1973). There exist several factors, for example, customer perceived value, trust, accessibility, and service quality that directly and positively influence satisfaction in diverse sectors (like the tourism industry, home delivery service, public transportation, and B2C e-commerce) (González-Rodríguez et al., 2019; Ghorbanzadeh et al., 2020; Rodríguez et al., 2020; Uzir et al., 2021).

Regarding music streaming services, satisfaction is the evaluative belief that customers have the choice to use and buy music being streamed on websites or apps they consider reputable, they deem the right choice, and where they believe that the service provided fulfills their needs (Cronin et al., 2000; Khatib et al., 2019). Based on previous literature, in streaming services, positive emotions, and customer engagement can influence consumers' satisfaction levels (Gupta and Singharia, 2021; Hsu et al., 2021). Also, Khatib et al. (2019) revealed that reliability and responsiveness, expressed as performance were significant to satisfaction (Khatib et al., 2019).

### Loyalty

Loyalty represents the extent to which a customer intends to continue using a particular information service (Bolton and Lemon, 1999). Essentially, two approaches are used in studies on loyalty where the behavioral approach theorizes loyalty as behavior, and consumers are considered loyal if they systematically purchase products or services over a certain period (Suhartanto et al., 2019). In music streaming services, behavioral loyalty is commonly measured by the frequency of using music streaming platforms. The second approach to studying loyalty involves using the attitudinal approach, which means loyalty intention or conative loyalty (Almeida-Santana and Moreno-Gil, 2018). Loyalty intention is the state of a customer's strong commitment to purchasing a product or service (Cong, 2016). However, Nguyen-Phuoc et al. (2021) suggest that loyalty does not refer to the willingness to purchase but observable behaviors, for example, the intention to recommend and repurchase. As a result, for streaming media services, loyalty can be seen as a user's intention to keep and order streaming services in the future (Gupta and Singharia, 2021).

### Time pressure

Most studies on time pressure can be divided into two groups: (1) time pressure as a situational variable impacting people's decision-making. In a working environment, time pressure affects professionals' judgment and decision-making processes (Ewing and Spilker, 2021). Also, time pressure affects consumers' purchase decisions in a shopping environment (Lin and Chen, 2013; Sohn and Lee, 2016). It more likely reflects a time constraint and deadline that induces some sense of stress and creates the need to deal with limited time (Svanström, 2016; Rostami et al., 2019; Bjorvatn and Wald, 2020). (2) Defining time pressure as a feeling or awareness that there is too much to do and not enough time in which to do it (Kleiner, 2014). The feeling of time pressure is of greatest concern theoretically and practically when it becomes a feature of daily experience and occurs frequently (Kleiner, 2014). In addition, it is connected to higher contracted time, for example, employment and school, and committed time, such as domestic labor or volunteering; therefore, it is more likely that people with larger schedules will feel higher time pressure (Haworth and Lewis, 2005; Hilbrecht et al., 2007).

Although many studies have adopted the situational variable approach to time pressure within a working environment or consumption situation (Lin and Chen, 2013; Sohn and Lee, 2016; Rostami et al., 2019), there is little research on time pressure as a factor that may affect the relationship between consumers' QSE and satisfaction, especially in the field of music streaming services. Time pressure refers to the sense of having insufficient time to finish things and being constantly rushed, which affects people's decision-making. Being under high time pressure boosts the impulsive purchase of hedonic products or services, while low time pressure increases the impulsive purchase of utilitarian products or services (Liu et al., 2022). The music streaming service provided by a platform constitutes an environment that contains hedonics. In general, consumers facing time pressure might be used to focusing on and be better able to quickly attain certain information types, thus leading to a greater likelihood of their use as inputs to choose (Nowlis, 1995). There is a controversy about the effect of time pressure on satisfaction (Skallerud et al., 2009; Nilsson et al., 2017). In addition, music is different from general commodities, and music listening could help people relax and reduce work-related stress (Raglio et al., 2020). However, there is little research on consumers' satisfaction with music streaming services under time pressure.

Furthermore, limited buying time reduces the number of alternatives considered. Increasing time pressure makes consumers more inclined to choose well-known brands (Mothersbaugh and Hawkins, 2015). In a food and grocery shopping environment, consumers under time pressure are more loyal to a favored store where they may shop with the least energy and time (Kongarchapatara and Shannon, 2016). There is little research on whether consumer satisfaction affects loyalty under time pressure. In this research, we expect time pressure to influence the relationship between QSE and satisfaction.

The second definition mentioned above is adopted in this research, with time pressure defined as the sense of having insufficient time to finish things and being constantly rushed (Hilbrecht et al., 2007).

### Hypotheses

The literature review illustrates variable correlations treated in this study, their complexities, and varying conceptualizations. This paper proposes a different method where QSE is considered a formative measurement of the consumers' assessments of their experience, and QSE is connected to satisfaction and loyalty. This section is guided by previous literature and seeks to support a set of hypotheses by describing past studies on the linkages between the variables investigated.

#### The impact of QSE on satisfaction and loyalty

Numerous studies used various theories to support factors influencing consumer satisfaction and willingness to consume and subscribe (or loyalty) to music streaming services. Bolduc and Kinnally (2018) used the original theory of planned behavior (TPB) and expanded TPB models and supported that they could both be employed in the context of digital music streaming use (Bolduc and Kinnally, 2018). Concretely, attitudes and social interaction positively contributed to the intention to use music streaming services (Kinnally and Bolduc, 2020). Barata and Coelho (2021) identified and examined the unified theory of acceptance and use of technology (UTAUT) created by Venkatesh et al. (2003) and its extension, UTAUT2, proposed by Venkatesh et al. (2012); that is, the willingness to pay for music streaming services is influenced by performance expectancy (favorable for consumers to perform certain activities); effort expectations (related to consumers' use of technology); hedonic motivation (pleasure or thrill derived from using a technology); price value (the perceived trade-off between the perceived benefit and monetary cost of the application to consumers); habit; perceived freemium-premium fit; involvement and interest; and attitude toward piracy (Barata and Coelho, 2021; Lüders, 2021). Also, Sinclair and Tinson (2017) and Danckwerts and Kenning (2019) proposed that music-based-psychological ownership is associated with users' willingness to choose paid services by using the psychological ownership theory improved by Pierce et al. (2003). Hsu et al. (2021) used the stimulus-organism-response theory (developed by Mehrabian and Russell, 1974) to propose that consumption emotion might only influence purchase intention by mediating consumer satisfaction, but cannot directly predict purchase intention (Hsu et al., 2021). Chang et al. (2021) used the theory of streaming service acceptance and proposed that attitudes, injunctive norms, and perceived behavioral control, except descriptive norms, can predict people's intentions to subscribe to premium music streaming services (Chang et al., 2021). However, there is a controversy about the effect of personalization. In this regard, Khatib et al. (2019), unlike Hsu et al. (2021) and Webster (2021), proposed that personalization positively influenced re-purchase intention, while previous studies revealed that QSE impacts users' satisfaction and intentions to keep and order streaming services in the future (Sackl et al., 2012; Gupta and Singharia, 2021).

Based on the development of the theory of QSE, this study chooses four dimensions of QSE hedonics, peace of mind, involvement, and recognition, and the following hypotheses are proposed:

H1: QSE positively and significantly impacts users' satisfaction with music streaming services.
H2: QSE positively and significantly impacts users' loyalty to music streaming services.

#### Satisfaction and loyalty

In the field of music streaming services, loyalty is influenced by customer engagement (Gupta and Singharia, 2021; Vinerean and Opreana, 2021). Also, there is evidence that satisfaction positively affects loyalty (Dai and Salam, 2019; Rodríguez et al., 2020). Therefore, the following hypothesis is proposed:

H3: Satisfaction has a direct positive and significant impact on users' loyalty to music streaming services.

Presenting the perspective that satisfaction from the previous experience is necessary for continued future purchases, different studies have examined satisfaction as a mediator that influences the relation among various constructs, including customer engagement, quality of service, emotion, service consumption experience, performance, behavior, willingness to continue and subscribe, purchase intention, loyalty, and re-purchase intention (Dai and Salam, 2019; Gupta and Singharia, 2021; Hsu et al., 2021). Therefore, the following hypothesis is proposed:

H4: Satisfaction mediates the relation between QSE and loyalty to music streaming services.

#### Time pressure as a moderator

**Figure 1:**
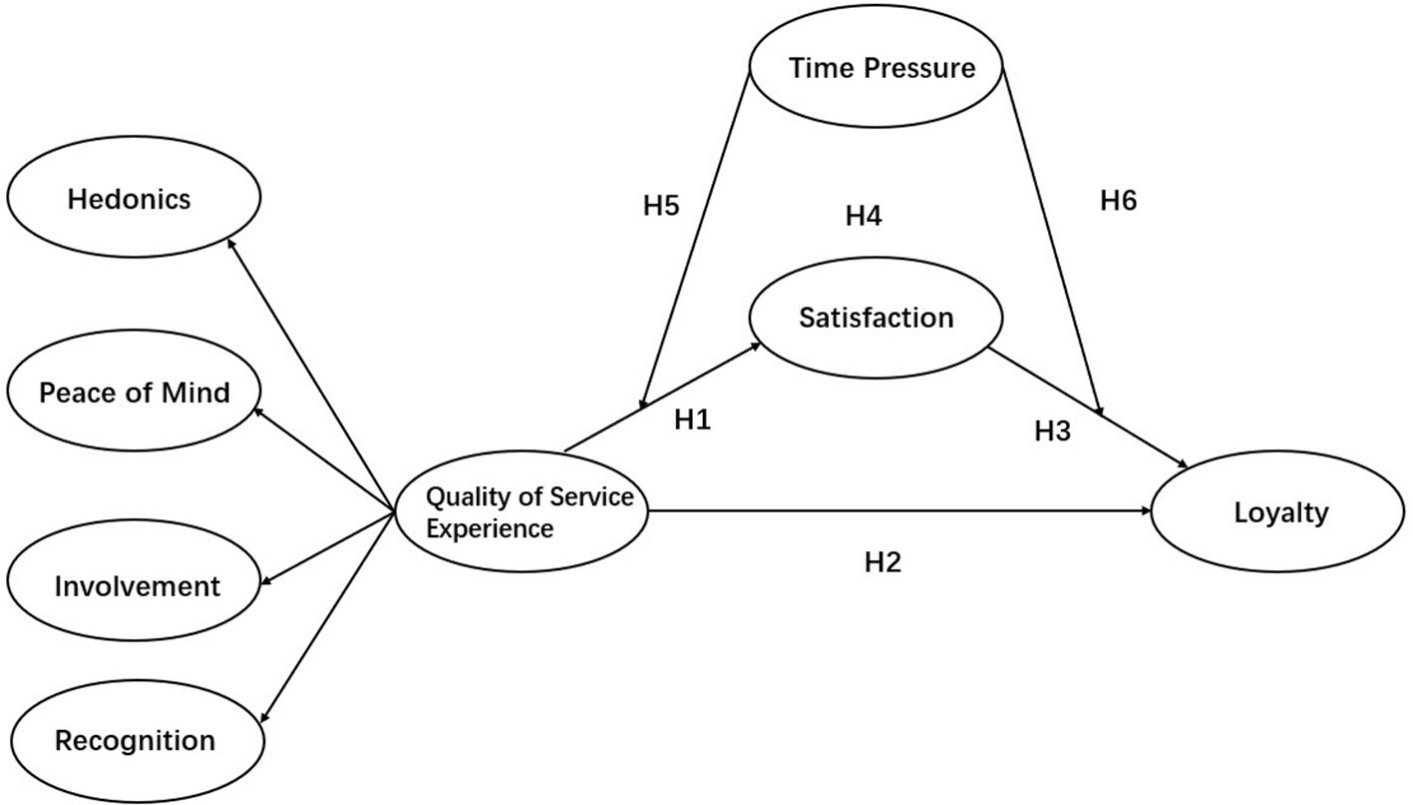
Proposed model.

Time pressure as a moderator has been discretely implied in several studies. For instance, within a shopping environment, time pressure may affect the relationship between emotions and impulse buying as a moderator (Sohn and Lee, 2016) and moderate the relationship between shopping motivations and commercial activities (Lin and Chen, 2013). Additionally, in a working environment, time pressure moderates the relationship between client preference and information research (Ewing and Spilker, 2021). In summary, time pressure shows a moderating effect in cases where the independent variable influences the dependent variable. However, there is little research to study time pressure as a feeling that moderates both the relationship between QSE and satisfaction and the relationship between satisfaction and loyalty. Therefore, the following hypotheses are proposed:

H5: Time pressure moderates the relationship between QSE and satisfaction with music streaming services.
H6: Time pressure moderates the relationship between satisfaction and loyalty to music streaming services. The proposed model is shown in Figure 1.

## Research method

This study aims to test QSE in music streaming, considering four dimensions (hedonics, peace of mind, involvement, and recognition) that affect loyalty and satisfaction with time pressure as a moderator using a quantitative method based on an online survey in China.

### Data collection and samples

In seeking to fulfill the study's objective, data was acquired by recruiting respondents through the online survey method. Participation in the study was both voluntary and confidential. Furthermore, to collect samples of respondents who use music streaming services, a question was asked, “Are you using music streaming services now?”. If the answer was “no”, the survey was immediately stopped. If the answer was “yes”, the survey would continue. The data collection period was over 2 months (January–February 2022), but participants' responses were analyzed collectively. A total of 341 participants completed the survey. After deleting inconsistent responses or extreme multivariate outliers, 267 usable samples were left. Outliers were excluded by using the graphic method, with a residual scatter plot in the range of ±3 standard deviations (Hair et al., 2010). The detailed information of the samples is shown in Table 1, reflecting 125 female and 142 male respondents. The age distribution is as follows: the largest group was aged 31- 40, comprising 128 respondents, followed by those aged 21–30 (92), and those aged over 41 (47). On careers, 110 respondents worked in private enterprises, comprising the largest group. The highest level of income distribution was 4,001–8,000 yuan, with 181 respondents in this income bracket.

**Table 1:** Demographics of respondents.

**Characteristics**	**Number (*N* = 267)**	**Percentage (%)**
**Gender**		
Male	142	53.2
Female	125	46.8
**Age**		
21–30	92	47.9
31–40	128	10.5
41–50	28	7.1
51–60	19	7.5
**Career**		
Student	11	4.1
State-owned enterprise	71	26.6
Public institution	30	11.2
Public servant	10	3.7
Private enterprise	110	41.2
Foreign enterprise	35	13.1
**Income**		
Less than 2,000 Yuan	20	7.5
2,001–4,000 Yuan	22	8.2
4,001–6,000 Yuan	90	33.7
6,001–8,000 Yuan	91	34.1
More than 8,000 Yuan	44	16.5

### Measures

In this study, the research items (listed in the Appendix) were drawn from prior research to emphasize the quality and validity of the survey instrument. Contents of all items required participants to indicate their level of agreement using a seven-point Likert scale (1 = strongly disagree, 7 = strongly agree). According to Otto and Ritchie's (1996) study, the construct measures tested in the questionnaire are as follows: 18 items measured four dimensions of QSE, which was also used previously by Cervera-Taulet et al. (2019) and Schlesinger et al. (2020); five items measured “satisfaction” based on the scale items proposed by Uzir et al. (2021); seven items were utilized to assess “loyalty”, according to existing measures applied by Hwang et al. (2019) and Nguyen-Phuoc et al. (2021), and five items measured “time pressure” extracted from the studies developed by Amiruddin (2019). Adopting scale items from previous research further enhanced this study's reliability. In addition, item expression was refined to indicate the scope of the study. Table 2 provides the measurement items for all constructs. Other demographic information was also included in the survey. Before this quantitative study, the survey instrument was pretested on 47 participants, and Cronbach's α was assessed. In this regard, the pretest results indicated high reliability of the scales.

**Table 2:** Constructs and corresponding items, loadings, and reliability scores.

**Constructs**	**Indicators**	**Factor loadings**	**T-value**	**Cronbach'α**	**CR**	**AVE**
HED	HED1	0.759		0.832	0.833	0.555
–	HED2	0.745	11.53^***^			
	HED3	0.736	11.395^***^			
	HED5	0.740	11.467^***^			
PM	PM1	0.657		0.794	0.801	0.575
	PM2	0.820	10.466^***^			
	PM3	0.788	10.266^***^			
INV	INV1	0.823		0.829	0.834	0.626
	INV4	0.796	13.535^***^			
	INV5	0.754	12.76^***^			
QSE	HED	0.792		0.797	0.865	0.681
	PM	0.807	7.573^***^			
	INV	0.874	8.886^***^			
SAT	SAT1	0.766		0.792	0.808	0.585
	SAT2	0.822	11.602^***^			
	SAT3	0.702	10.537^***^			
LOY	LOY1	0.754		0.856	0.857	0.600
	LOY2	0.811	12.755^***^			
	LOY3	0.724	11.404^***^			
	LOY5	0.805	12.673^***^			
TPR	TPR1	0.817		0.853	0.855	0.663
	TPR3	0.856	13.581^***^			
	TPR5	0.768	12.815^***^			

AVE, average variance extracted.

*** Significant at the 1% level.

This study adopted a two-step data analysis procedure and structural equation modeling (SEM) proposed by Anderson and Gerbing (1988). Specifically, the first step concentrates on examining the outer, or measurement model to evaluate construct reliability, convergent validity, and discriminant validity. In the second step, the inner model, namely the structural model, is tested to analyze the relationships between the independent and dependent variables. These tests used maximum likelihood estimation using AMOS 16.0.

## Results

### Measurement model

Confirmatory factor analysis was conducted to examine the measurement model's reliability, convergent validity, and discriminant validity. The results in Table 2 illustrate that Cronbach's α for all constructs was higher than 0.7 (Hair et al., 2010). Hair et al. (2010) suggested that the composite reliability should be larger than 0.7, and the composite reliability of all constructs was within the range of 0.801 and 0.865, identical to Hair et al. (2010), and displayed sufficient reliability. The average variance extracted (AVE)s for each construction (between 0.555 and 0.681) was greater than the recommended level of 0.5 (Fornell and Larcker, 1981). Therefore, the convergent validity of all constructs was established. All the standardized factor loadings were higher than the required threshold of 0.6 (Hair et al., 2010), except for three items (HED4, HED6, and HED7) for the construct “hedonics” (HED), one item (PM4) for the construct “peace of mind” (PM), two items (INV2 and INV3) for the construct “involvement” (INV), two items (SAT4 and SAT5) for the construct “satisfaction” (SAT), three items (LOY4, LOY6, and LOY7) for the construct “loyalty”(LOY), and two items (TPR2 and TPR4) for the construct “time pressure” (TPR). At the same time, two-order confirmatory factor analysis was applied for the construct QSE (Doll et al., 1994). All instances of composite reliability were above the required threshold of 0.7, except the values for construct “recognition” (REC), which were <0.7 and deleted. The variance and residuals of leftover constructs were positive and significant, which did not offend the estimate (Hair et al., 2010).

After deleting the construct recognition, the results in Table 3 show that the two-order three-factor model and one-order three-factor (correlated) model were equivalent because both had an identical model fit. However, the model fit of the two-order three-factor model was better than the null model, the three first-order factor (uncorrelated) model, and the one first-order factor model. This outcome indicates that three constructs (hedonics, peace of mind, and involvement) can be used for the two-order factor model, quality of service experience.

**Table 3:** Two order CFA and fit index of QSE.

**Two order CFA model of QSE**	**χ2**	**df**	**χ2/df**	**GFI**	**AGFI**	**NFI**	**CFI**	**RMSEA**
Null Model	1258.200	45	27.960	0.348	0.203	0	0	0.318
Three-first factors (uncorrelated) model	302.291	35	8.637	0.810	0.701	0.760	0.780	0.169
First-order factor model	260.739	35	7.450	0.812	0.704	0.793	0.814	0.156
Three-first factors (correlated) model	63.511	32	1.985	0.952	0.917	0.950	0.974	0.061
Three-second factors (correlated) model	63.511	32	1.985	0.952	0.917	0.950	0.974	0.061
Recommended value	Less	Larger	<3	>0.8	>0.8	>0.9	>0.9	<0.08

The constructs' discriminant validity was evaluated utilizing the criterion put forward by Fornell and Larcker (1981). As shown in Table 4, for each construct, the square root of the AVE was higher than its correlation with any other construct. Correlations among all constructs were obviously smaller than 0.9 (Hair et al., 2010), suggesting that multicollinearity is not likely to be a problem in this study.

**Table 4:** Discriminate validity examination.

	**Mean**	**SD**	**QSE**	**TPR**	**LOY**	**SAT**
QSE	4.517	0.970	**0.825**			
TPR	3.880	1.564	−0.230	**0.814**		
LOY	5.141	1.140	0.597	−0.159	**0.774**	
SAT	3.042	1.134	0.560	−0.152	0.543	**0.765**

The bold diagonal elements are the square roots of each AVE; construct correlations are shown off-diagonal.

As shown in Table 5, the overall measurement fit indices indicate that the confirmatory factor model fits the data well. For the measurement model, $\frac{\chi^{2}}{df}=1.832$ was smaller than three, SRMR was 0.055, RMSEA was 0.056, GFI was 0.898, and AGFI was 0.867, which were acceptable (Doll et al., 1994; Tabachnick and Fidell, 2013). Also, IFI was 0.948, CFI was 0.947, and TLI was 0.938, all larger than the suggested value of 0.9 (Hooper et al., 2008; Hair et al., 2010). Therefore, this outcome indicates that the measurement model was acceptable.

**Table 5:** Fit indices of measurement and structural model.

**Fit induces**	**χ^2^/df**	**SRMR**	**RMSEA**	**GFI**	**AGFI**	**IFI**	**CFI**	**TLI**
Measurement model	1.832	0.055	0.056	0.898	0.867	0.948	0.947	0.938
Structural model	1.792	0.050	0.055	0.914	0.884	0.959	0.958	0.950

### Structural model

Structural equation modeling was introduced to examine the hypothesized relations among variables, employing maximum likelihood estimation. As shown in Table 5, the goodness-of-fit statistics of the structural models provide a well-fitting model ($\frac{\chi^{2}}{df}=1.832$; SRMR = 0.55; GFI = 0.914; AGFI = 0.884; IFI = 0.959; CFI = 0.958; TLI = 0.950) (Blunch, 2008; Hair et al., 2010).

#### Direct effects

**Figure 2:**
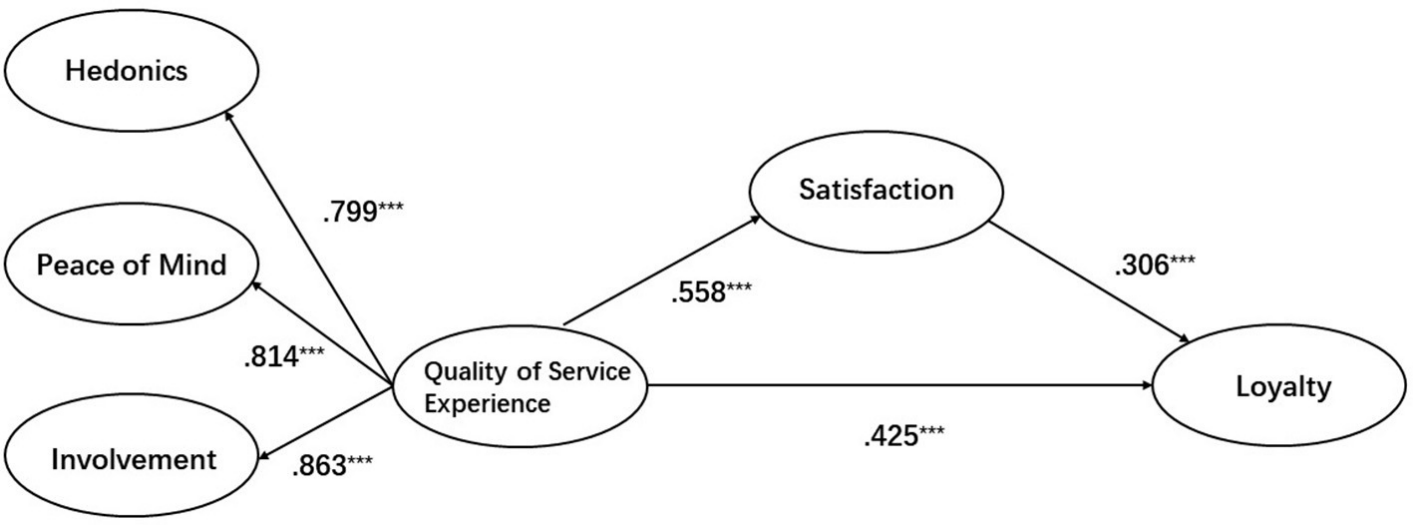
The results of path analysis.

Regarding the model, the analysis provides evidence shown in Table 6 that QSE (β = 0.522, *t* = 6.429, *P* < 0.001) was positively related to satisfaction, supporting H1. Moreover, we found that QSE (β = 0.495, *t* = 4.694, *P* < 0.001) was positively related to loyalty, supporting H2. The results also reveal a relationship between satisfaction and loyalty (β = 0.381, *t* = 3.675, *P* < 0.001). Thus, H3 was supported. Figure 2 depicts the result of the path analysis.

**Table 6:** Hypothesized relation.

**Hypothesized relation**		**Unstd**.	**S.E**.	***t*** **-value**	**sig**.	**Std**.	**Supported?**
H1	QSE → SAT	0.522	0.081	6.429	^***^	0.558	Supported
H2	QSE → LOY	0.495	0.105	4.694	^***^	0.425	Supported
H3	SAT → LOY	0.381	0.104	3.675	^***^	0.306	Supported

*** Significant at the 1% level.

#### Mediating effects of satisfaction

Although the Baron and Kenny method (Baron and Kenny, 1986) and Sobel test (Sobel, 1982, 1986) are widely applied for testing mediators, Hayes (2009) proposed bootstrapping as a better choice. In this study, to assess whether satisfaction mediates the relation between QSE and loyalty, percentage bootstrapping and bias-corrected percentage bootstrapping with a bootstrap sample of 1,000 at a 95% confidence interval were conducted according to the suggestion in Taylor et al. (2008). Results in Table 7 indicate that the indirect effect of QSE on loyalty (LOY) through satisfaction (SAT) is significant (point estimate = 0.199, SE = 0.066, Z = 3.015, bias-corrected: 0.087–0.354, percentage: 0.070–0.337), so H4 was supported. In addition, the direct effect of QSE on loyalty (LOY) and the total effect was also significant.

**Table 7:** Bootstrapping mediating effect testing.

**Path relation**	**Point estimate**	**Product of coefficient**		**Bootstrapping 1,000 times 95% CI**			
				**Bias-corrected**		**Percentile**	
		**SE**	**Z**	**Lower**	**Upper**	**Lower**	**Upper**
Indirect effect							
QSE → SAT → LOY	0.199	0.066	3.015	0.087	0.354	0.070	0.337
Direct effect							
QSE → LOY	0.495	0.135	3.667	0.230	0.767	0.233	0.769
Total effect							
QSE → LOY	0.694	0.124	5.597	0.459	0.959	0.456	0.951

#### Moderated mediation effects of time pressure

The proposed moderator satisfaction (SAT) was incorporated into the model to study the fully specified moderated mediation model. For this purpose, PROCESS v.3.5 Model 58, created by Hayes (2018), was conducted. This technique uses percentage bootstrapping and bias-corrected percentage bootstrapping with a bootstrap sample of 1,000 at 95% confidence intervals, providing findings for moderation and conditional indirect effects between the independent and the dependent variable through the mediator at various levels of moderation. Thus, Model 58 of PROCESS provides the assessment of fully moderated mediation.

As evident from Table 8, the influence of the interaction term (between QSE and time pressure) on satisfaction has a coefficient of 0.097, and a *p*-value is smaller than 0.05. Therefore, the moderating effect is significant. Additionally, the influence of the interaction term (between satisfaction and time pressure) on loyalty has a coefficient of 0.084, and a *p*-value is smaller than 0.05. Hence the moderating effect is significant. These results are in line with H5 and H6.

**Table 8:** Moderated mediation model testing.

**Antecedent**	**Consequent**					
	**SAT (M)**			**LOY(Y)**		
	**Coeff**.	**SE**	***p***	**Coeff**.	**SE**	***p***
Constant	2.861	0.824	0.001	3.675	0.627	0.000
QSE(X)	0.095	0.174	0.585	0.368	0.071	0.000
SAT(M)	–	–	–	−0.012	0.144	0.935
TPR(W)	−0.497	0.176	0.005	−0.290	0.119	0.016
X × W	0.097	0.038	0.010	–	–	–
M × W	–	–	–	0.084	0.036	0.021
	R2 = 0.228			R2 = 0.332		
	F = 25.953, *p* < 0.001			F = 32.548, *p* < 0.001		

X: independent variable; M: Mediator; W: Moderator; Y: dependent variable.

**Figure 3:**
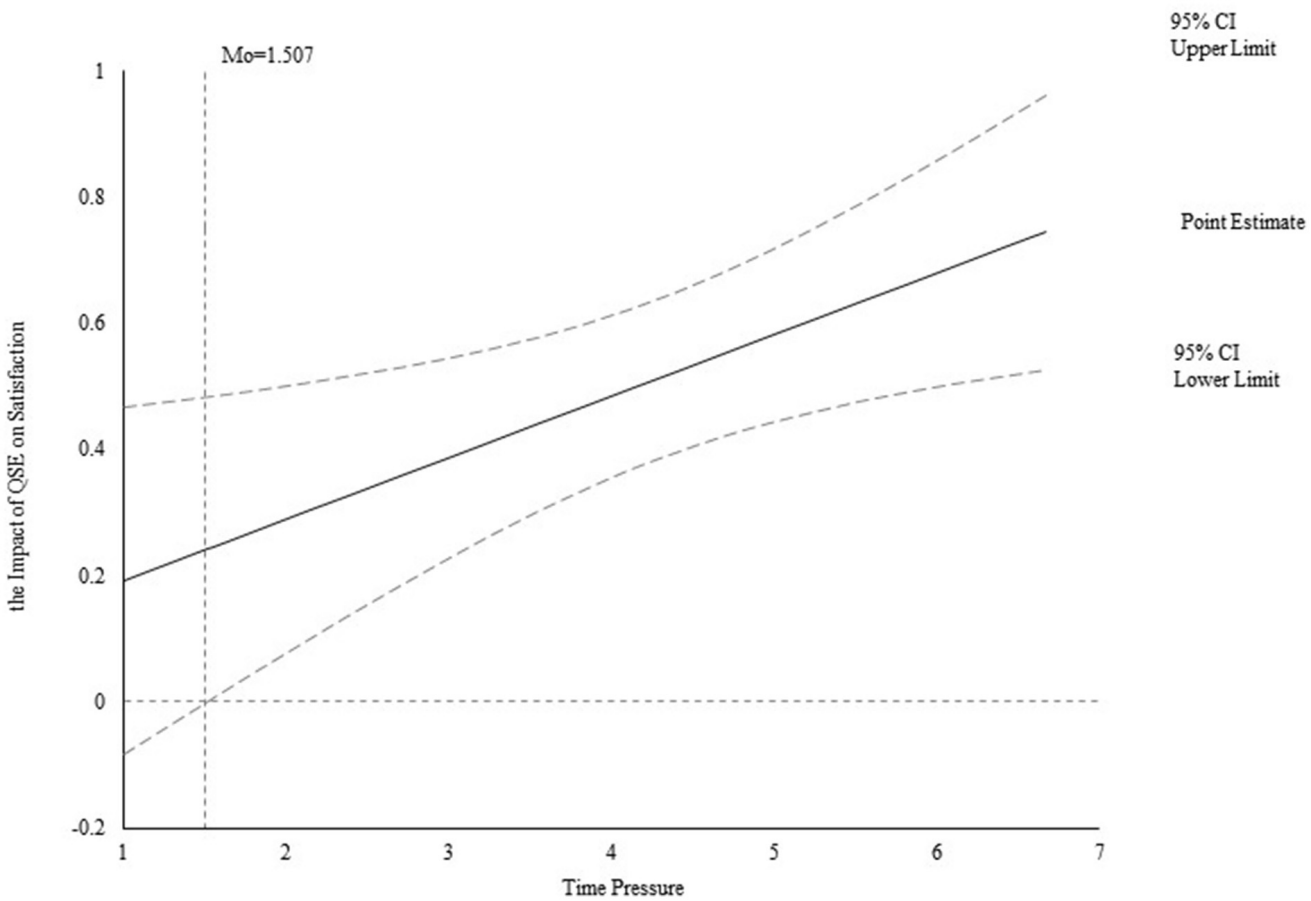
Effect of time pressure on the relation between QSE and satisfaction.

**Figure 4:**
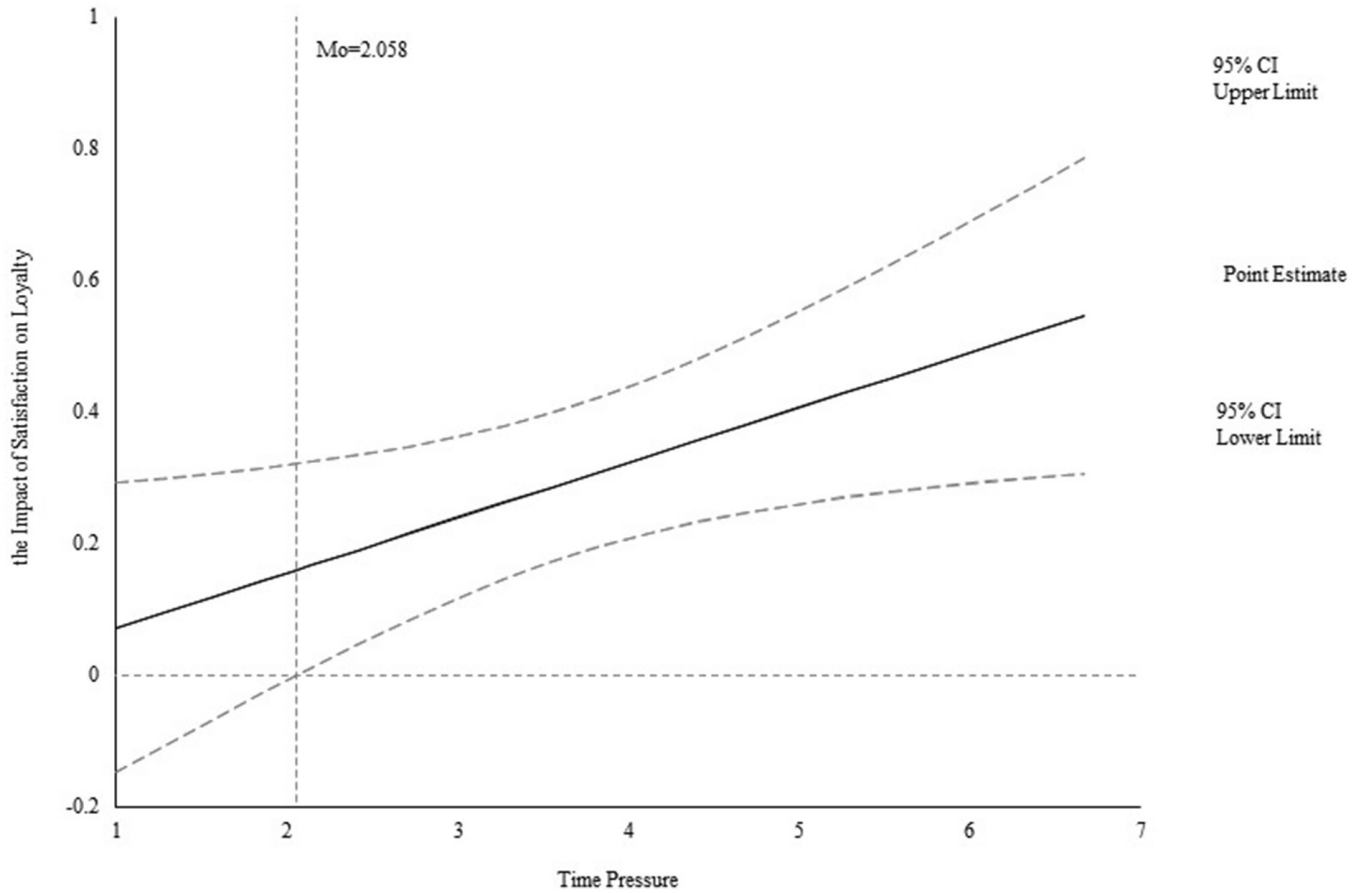
Effect of time pressure on the relation between satisfaction and loyalty.

Furthermore, Spiller et al. (2013) advised using the Jonson-Neyman technique to measure the moderating effect to detect moderating intervals. This technique identifies the level from which the moderator begins to have a moderating effect between the independent and the dependent variable. The study accesses statistically significant intervals of moderating effects by measuring the effect of QSE on satisfaction when time pressure has different values and tests the impact of QSE on satisfaction. As seen in Figure 3, the moderating effect is positively significant when the value of time pressure is larger than 1.507 but the moderating effect is insignificant when the value is smaller than 1.507. In addition, Figure 4 confirms that the moderating effect is positively significant when the value of time pressure is larger than 2.058, but the moderating effect is insignificant when the value is smaller than 2.058. Both results indicate the greater positive effect of customers' satisfaction on loyalty with the higher level of time pressure.

A bootstrapping test is conducted in the mediation model to test the moderated indirect effect of time pressure on satisfaction. As seen in Table 9, with a bootstrap sample of 1,000 at 95% confidence intervals, when time pressure is low (TPR = Mean – 1SD), the indirect effect of QSE on loyalty through satisfaction is insignificant (including zero in Boot 95% CI intervals). Second, when time pressure is medium (TPR = Mean), the conditional indirect effect of QSE on loyalty through satisfaction is significant (Effect = 0.148, and Boot 95% CI intervals do not include zero). Then, when time pressure is high (TPR = Mean + 1SD), the indirect effect of QSE on loyalty through satisfaction is significant (Effect = 0.277, and Boot 95% CI intervals do not include 0). At the same time, when time pressure changes to low, medium, and high, the indirect effect of QSE on loyalty through satisfaction is significant (Boot 95% CI intervals do not include zero). In addition, using the method of testing moderated indirect effects, the direct effect in moderated mediation model is significant (direct effect = 0.368) as bootstrap 95% confidence intervals are [0.229, 0.507] not including 0. Therefore, these results support the moderated-mediation pattern between QSE and loyalty when time pressure is a moderator.

**Table 9:** Bootstrapping test of moderated indirect effects.

**Results**	**TPR**	**Effect**	**BootSE**	**BootLLCI**	**BootULCI**
Moderated Indirect Effects	Low (M-1SD)	0.058	0.034	−0.004	0.132
	Medium (M)	0.148	0.037	0.082	0.237
	High (M+1SD)	0.277	0.068	0.150	0.416
Comparison of effects	Medium–Low	0.090	0.027	0.040	0.147
	High–Low	0.219	0.074	0.075	0.365
	High–Medium	0.130	0.048	0.038	0.224

## Discussion and implications

This study aimed to examine the effect of QSE on consumers' satisfaction and loyalty to music streaming. For this, the conceptualization of the QSE construct in music streaming services was introduced. Also, the present validated instrument for this study was presented. Then, convergent and discriminant validities were presented to facilitate an evaluation of the latent structure of the QSE as a second-order factor. Three first-order formative dimensions of the QSE were determined and verified: hedonics, peace of mind, and involvement, while recognition was deleted as the composite reliability value was <0.7 (Doll et al., 1994). Then, based on the theory of QSE created and developed by Otto and Ritchie (1996), the relationship between satisfaction and QSE and the relation between loyalty and QSE were examined. Furthermore, the relationship between satisfaction and loyalty was investigated, assessing the effect of QSE on loyalty through satisfaction. Additionally, this study examined the influence of QSE on consumer satisfaction with feelings of time pressure due to work and the influence of satisfaction on consumers' willingness to continue subscribing to music streaming services (albeit loyalty) while facing work-induced time pressure. Regarding the antecedents (dimensions), the SEM results reveal that QSE positively influences both satisfaction and loyalty. In addition, QSE impacts loyalty through satisfaction when satisfaction positively influences loyalty. The results further indicate that a higher level of satisfaction and loyalty with a higher level of time pressure. Hence, music streaming that enhances consumers' willingness to continue their subscriptions should address QSE including hedonics, peace of mind, and involvement. These findings support the suggestion by Cervera-Taulet et al. (2019) that QSE might be the most effective route for stimulating repeat use.

The results indicate a strong relationship between QSE and satisfaction (H1), being consistent with existing research dedicated to QSE in digital content such as OTT media streaming (Gupta and Singharia, 2021) and creative tourism (Suhartanto et al., 2019). Users show satisfaction with music streaming services by feeling hedonic, having peace of mind, and being involved when they enjoy music and use social functions such as commenting and sharing music from music streaming services. However, this contradicts Dai and Salam's (2019) study, supporting an insignificant relationship between consumption and satisfaction in electronically mediated environments. In addition, the results determined QSE as an antecedent of users' loyalty (H2). Users indicated a higher willingness to continue subscribing to music streaming services when feeling higher levels of QSE. This outcome supports the research findings by Gupta and Singharia (2021) who determine QSE as an antecedent of loyalty when using OTT media streaming services through a first-order construct for QSE. Therefore, providers of music streaming services should enhance recreational functions for users to entertain themselves instantly and conveniently, as most consumers access music streaming services through smartphones. Specifically, providers are advised to encourage users to create or participate in some virtual communities *via* platforms and comment and express their thoughts about music with others. Also, providers should encourage users to share their favorite songs with their friends through music streaming platforms and other social media platforms. To this end, strengthening cross-platform cooperation is advised. In addition, as users feel peace of mind and mental relaxation when they enjoy music, providers should choose high-quality songs and classify them with some tags such as “peace”, “quiet”, and “happiness” and carry no advertisements in these classifying playlists because advertisements negatively affect users' intention (Mishra and Malhotra, 2020). In other words, advertisements could distract consumers from listening to music, reducing favorability, and a simple interface will help people relax, allowing them to concentrate on enjoying music (Cyr et al., 2006). Moreover, it is suggested that users' sense of involvement could be enhanced by encouraging them to participate in music streaming platform construction and collecting their advice to enhance functionality and optimize the interface.

As predicted, the results also indicate that satisfaction leads to loyalty in using music streaming services (H3). These are consistent with the suggestion of Dai and Salam (2019) in the case of electronic-mediated environments and the findings by Khatib et al. (2019) in their research on millennial streaming service users. Furthermore, the results reveal that higher satisfaction leads to higher intentions to keep and order music streaming services. Furthermore, satisfaction mediated the relation between QSE and loyalty (H4), although the percentage of direct effect (QSE → LOY) is larger than the percentage of indirect effect (QSE → SAT → LOY). The result is consistent with previous studies that have shown that marketers of streaming services should pay attention to user satisfaction (Gupta and Singharia, 2021). The results further reveal that satisfying users' spiritual needs, such as socializing, expressing themselves, and having a mental release will lead them to continue paying for the subscription discussed in H2. In other words, music streaming service subscribers are concerned with the platform's content that satisfies their spiritual needs. These spiritual needs come from the diverse content and activities the music streaming platform provides, for instance, the quantity of music, the quality of music, and activities related to the virtual community. Therefore, it is suggested that providers focus on the quality of the content and develop various activities that will allow users to actively engage in them for a long time or simply enjoy the music.

Our empirical analysis supports our assumption that the interaction between QSE and time pressure is linked to users' satisfaction with music streaming services (H5). We find a higher level of influence of QSE on satisfaction with services offered by music streaming platforms at higher levels of time pressure, which is consistent with Skallerud et al. (2009). In other words, consumers relax and experience more positive emotions by listening to music that meets their expectations, so they tend to be more satisfied with music streaming services. Thus, the influence of music on people's emotions and the influence of emotions on satisfaction cannot be ignored. In addition, the result reveals that people tend to seek some “fun” to release nervousness when facing time pressure. Therefore, users will have good feelings and higher satisfaction with the methods and channels they use when they perceive that their stress is perfectly released. Consequently, to recommend appropriate music variation and virtual community activities that match users' conditions, providers should understand the current psychological state of users through surveys within the platform and, with users' permission, track their recent music listening or song lists. Furthermore, it is vital to understand that users could release their stress by participating in various activities and enjoying music on music streaming platforms, which can enhance their satisfaction with music streaming services. In other words, providers should focus on consumers' behavior in using music streaming services.

As predicted, the results also show that the interaction between time pressure and satisfaction is linked to users' loyalty (H6). This interaction reveals that for users of music streaming services, satisfaction has a greater impact on loyalty under greater time pressure, and they are more willing to continue subscribing given the greater time pressure, which is consistent with findings about general commodities (Kongarchapatara and Shannon, 2016). This result extends previous research that discuss time pressure as an environmental factor that negatively influences staff judgment and decision-making (Santos and Cunha, 2021). The result is also somewhat consistent with previous research that identified time pressure during shopping as leading to increased affective impulse buying (Lin and Chen, 2013; Sohn and Lee, 2016). Additionally, given that people always prefer familiar environments and content, the previously mentioned result indicates that when people are familiar with a method for relieving stress, they will tend to resort to the same method they used in the past whenever they encounter a stressful situation. According to the theory of mere exposure, which is a psychological phenomenon, people tend to favor things or people that are more familiar to them than others (Zajonc, 1968). As a result, marketers should consider user satisfaction to maintain and improve the number of subscriber renewals.

From a theoretical perspective, this study makes several contributions to the marketing literature. First, our research provides evidence that QSE influences user satisfaction and loyalty to music streaming services, unlike the theory on QSE created by Otto and Ritchie (1996) which was limited to studying consumers in tourism. This study enriches the existing limited research concentrating on users' intention to pay for music streaming services and willingness to continue subscribing. Second, this study provides the first quantitative examination of how time pressure affects the relation between QSE and satisfaction and the relationship between satisfaction and loyalty for users of music streaming services. Consequently, this study contributes to previous research that revealed that time pressure affects people's purchase decisions in shopping environments.

## Limitations and further work

This study has limitations that can potentially offer direction for further research. Firstly, the difference in the geographical location of consumers is ignored, and geographic factors may lead to different results. China is a multinational country with a vast territory spanning four time zones. People in different regions may have different concepts of time. There is a difference in time conception, including from monochronic and poly-chronic time perspectives, which may lead to people having diverse perceptions of time (Mothersbaugh and Hawkins, 2015). An individual with a monochronic time perspective can only do one thing at a given time. Time pressure drives this group to eagerly complete their work at hand and not to take time to enjoy music. People with a multi-directional view of time can do multiple things, such as listening to songs while working. They will use enjoying music as a means of relieving the stress of their work. Therefore, the moderating effect of time pressure may be more pronounced in groups with a multidirectional view of time. Future research models could compare consumer decisions across domains to better understand consumers' QSE and willingness to continue subscribing to music streaming services. Additionally, as people from different cultural backgrounds may have various perceptions of time, it might be important to research from a global perspective in the future.

Secondly, the proposed conceptual model did not incorporate purchase behavior; however, there is a differentiation between the evaluation of the behavioral intention, such as loyalty, and the practice. Thus, further research could involve a longitudinal study to analyze the actual renewal action as cross-sectional data is applied to the study. Thirdly, this study examined the effect of work-induced time pressure in the field of music streaming services consumption. It seems worthwhile to apply the model to other digital content such as movies, digital books, and animation. The results may be differed depending on the type of streaming service.

## Conclusion

The study aimed to examine the relationship between QSE and user satisfaction and that between QSE and loyalty to music streaming platforms. According to the theory of QSE proposed by Otto and Ritchie (1996), the results indicated that better QSE leads to higher satisfaction and loyalty, highlighting the importance of QSE, such as in terms of hedonics, peace of mind, and involvement for providers of music streaming services. Our findings demonstrated that time pressure from working is positively related to the relationship between QSE and satisfaction, and the relationship between satisfaction and loyalty, revealing to marketers the importance of stress release through enjoying music and communicating with others on music streaming platforms.

## Data availability statement

The raw data supporting the conclusions of this article will be made available by the authors, without undue reservation.

## Ethics statement

Ethical review and approval was not required for the study on human participants in accordance with the local legislation and institutional requirements. Written informed consent from the [patients/ participants OR patients/participants legal guardian/next of kin] was not required to participate in this study in accordance with the national legislation and the institutional requirements.

## Author contributions

YZ: conceptualization and formal analysis and writing— review and editing. MZ: investigation. YZ and MZ: writing—original draft. Both authors contributed to the article and approved the submitted version.

## Conflict of interest

The authors declare that the research was conducted in the absence of any commercial or financial relationships that could be construed as a potential conflict of interest.

## Publisher's note

All claims expressed in this article are solely those of the authors and do not necessarily represent those of their affiliated organizations, or those of the publisher, the editors and the reviewers. Any product that may be evaluated in this article, or claim that may be made by its manufacturer, is not guaranteed or endorsed by the publisher.

## Appendix

**Table A1:** Measurement items.

**Construct**	**Items**	**References**
Quality of Service Experience (QSE)	Hedonics (HED): HED1. In this platform, I am doing something I really like to do. HED2. I am doing something memorable that enriches my life. HED3. This experience is exciting. HED4. I am having a “once in a lifetime” experience.^*^ HED5. I can share my experience with other. HED6. I am being challenged in some way.^*^ HED7. My imagination is being stirred.^*^ HED8. It feels like I am on an adventure. HED9. Using service (listening to the music, interacting with friends etc.) experience provides me fun. HED10. With this digital music platform, I established friendships with one or more new people. HED11. This digital music platform let me feel that I am doing something new and different. Peace of mind (PM): PM1. Using this platform let me feel physically comfortable. PM2. Using this platform let me feel that my property is safe. PM3. Using this platform let me feel a sense of personal security. PM4. Using this platform let me feel that my privacy is assured.^*^ Involvement (INV): INV1. I feel a sense of cooperation. INV2. I feel I am involved in the process of enhancing function.^*^ INV3. I think there is an element of choice in the process.^*^ INV4. I feel I have some control over the outcome. INV5. I think I am being educated and informed. Recognition (REC)^*^ REC1. I feel that I am being taken seriously. REC2. I feel that I am important.	Cervera-Taulet et al., 2019; Schlesinger et al., 2020
Satisfaction (SAT)	SAT1. The digital music platform service meets my expectations. SAT2. I am satisfied with my decision to use this digital music platform service. SAT3. I would avail their service the next time. SAT4. I will recommend others to use this digital music platform service.^*^ SAT5. I am very satisfied with the human-driven service.^*^	Uzir et al., 2021
Loyalty (LOY)	LOY1. I would tell other people positive things about this digital music service. LOY2. I would recommend other people to listen to music by this digital music platform service. LOY3. I would provide my friends, family, and neighbors with positive advice about this digital music service when they are choosing a mode for listening to the music. LOY4. I intend to listen to music by this digital music service more often in the future.^*^ LOY5. I feel better listening to music by this digital music service. LOY6. I prefer listening to music by this digital music service to others.^*^ LOY7. I intend to keep listening to music by this digital music service in the future.^*^	Hwang et al., 2019; Nguyen-Phuoc et al., 2021
Time Pressure (TPR)	TPR1. I only have limited time to finish task. TPR2. To reach time target, I must reduce my work.^*^ TPR3. I must shift time for different clients. TPR4. I must request additional time to finish work.^*^ TPR5. I have never reported actual time use.	Amiruddin, 2019

* Items deleted due to low loading.

Contents of all items required participants to indicate their level of agreement using a seven-point Likert scale (1 = strongly disagree, 7 = strongly agree).
